# Evaluation of Arabica Coffee Fermentation Using Machine Learning

**DOI:** 10.3390/foods13030454

**Published:** 2024-02-01

**Authors:** Renata A. R. Rocha, Marcelo A. D. da Cruz, Lívia C. F. Silva, Gisele X. R. Costa, Laurence R. Amaral, Pedro L. L. Bertarini, Matheus S. Gomes, Líbia D. Santos

**Affiliations:** 1 Biotechnology Institute, University Federal of Uberlândia, Patos de Minas 38700-002, MG, Brazil; renata.reis@ufu.br (R.A.R.R.); marcelo.antonio@ufu.br (M.A.D.d.C.); livia.fidelis@ufu.br (L.C.F.S.); 2 Faculty of Chemical Engineering, Federal University of Uberlândia, Patos de Minas 38702-178, MG, Brazil; gisele.ribeiro@ufu.br; 3 Laboratory of Bioinformatics and Molecular Analysis (LBAM), Federal University of Uberlândia, Patos de Minas 38702-178, MG, Brazil; laurence@ufu.br (L.R.A.); matheusgomes@ufu.br (M.S.G.); 4 Faculty of Electrical Engineering, Federal University of Uberlândia, Patos de Minas 38702-178, MG, Brazil; bertarini@ufu.br

**Keywords:** coffee fermentation, specialty coffees, sensory quality, coffee processing, coffee metabolites

## Abstract

This study explores the variances in the organic, chemical, and sensory attributes of fermented coffee beans, specifically examining how post-harvest processes influence cup quality. Coffee fruits from the Catuaí IAC-144 variety were processed using both natural coffee (NC) and pulped coffee (PC) methods. The fruits were then subjected to self-induced anaerobic fermentation (SIAF) using one of the following fermentation methods: solid-state fermentation (SSF) or submerged fermentation (SMF). Within these methods, either spontaneous fermentation (SPF) or starter culture fermentation (SCF) was applied. Each method was conducted over periods of 24, 48, and 72 h. For this purpose, two-hundred-liter bioreactors were used, along with two control treatments. Numerous parameters were monitored throughout the fermentation process. A comprehensive chemical profiling and sensory analysis, adhering to the guidelines of the Specialty Coffee Association, were conducted to evaluate the influence of these fermentation processes on the flavor, aroma, and body characteristics of the coffee beverage across multiple dimensions. Data analysis and predictive modeling were performed using machine learning techniques. This study found that NC exhibited a higher production of acids (citric, malic, succinic, and lactic) compared to PC, resulting in distinct chemical and sensory profiles. The decision tree showed that fructose and malic and succinic acids were identified as the main factors enhancing sensory notes during cupping. SMF promoted higher concentrations of lactic acid, while SSF led to increased ethanol content. Consequently, the SIAF process enhances the sensory quality of coffee, adding value to the product by generating diverse sensory profiles.

## 1. Introduction

Coffee is recognized as one of the world’s most heavily traded commodities. According to the International Coffee Organization, the production of Arabica coffee varieties reached 94.3 million bags in coffee year 2021/2022 [1]. Arabica coffee represents 56% of the global market and is predominantly utilized in the production of high-quality specialty coffees, known for their rich aroma and flavor [2,3]. The ultimate quality, gastronomic appeal, and economic value of a coffee product are closely linked to the complexity of flavors developed during the coffee bean processing phase, as highlighted in various studies [4,5].

In response to the specialty coffee market’s demand for coffees with distinct sensory profiles, producers are increasingly focusing on innovative post-harvest practices. These practices are aimed at elevating the quality and sensory uniqueness of their coffee offerings [6]. Over the years, post-harvest processes have undergone significant evolution. The implementation of controlled fermentation, particularly using starter cultures, has been identified as a promising approach for flavor enhancement, the generation of volatile compounds, and achieving product consistency. This approach is supported by various studies that demonstrate its effectiveness in augmenting the sensory appeal of coffee [6,7,8,9,10,11,12,13,14,15].

Coffee fermentation occurs naturally and involves of the degradation of the mucilage surrounding the beans by endogenous microorganisms naturally present in coffee [16]. During this stage, microorganisms metabolize sugars and form precursors of aroma and flavor, such as organic acids, esters, and alcohols, which directly influence the characteristics and flavors of the coffee [17,18].

This enhancement in sensory quality is attributed to alterations in the coffee bean’s chemical composition, significantly influenced by factors such as microorganisms, enzymatic reactions, and environmental parameters including temperature, the presence or absence of oxygen, and the duration of the process [19]. The microorganisms involved in coffee fermentation can be endophytic, originating from within the plant, or epiphytic, deriving from the surrounding environment of the fermentation container, such as soil, air, equipment, and people, as well as any other entities that make physical contact with the fruits [20]. The microbiota, in conjunction with the environmental conditions and process control at the farm, can either contribute to or detract from the production of high-quality coffee. Implementing appropriate processes that adhere to food safety and hygiene standards at all stages of processing can lead to improvements in quality [21].

Each stage of the post-harvest process, which invariably involves fermentation reactions, can significantly impact the sensory profile of the coffee [22]. In this context, variations in sensory profiles are achieved through the application of different fermentation methods. These methods can alter the chemical composition of coffee beans via the activity of microbial and/or endogenous enzymes present in the coffee [17,23,24].

The coffee fermentation process is a complex, multivariate system influenced by various environmental conditions, such as temperature, pH, water presence, and epiphytic microbiota. Additionally, characteristics intrinsic to the coffee fruit, like its variety and processing method (natural or pulped), also play a crucial role [7,10,14]. The variables in coffee processing can be categorized into four key stages or terms: the type of fruit treatment (natural or pulped), the environmental conditions (oxygen availability), the addition of water (solid-state fermentation—SSF or submerged fermentation—SMF), and the use of starter cultures [6].

Reflecting the diversity of processing variables, new fermentation techniques in coffee bean processing have emerged, such as self-induced anaerobic fermentation (SIAF). In SIAF, the coffee (either cherries or pulped) is placed in a closed container which serves as a bioreactor. The air inlet of the container is sealed, allowing the oxygen inside to be consumed by microorganisms and the fruit’s metabolism, leading to the production of carbon dioxide (CO_2_). This anaerobic environment fosters a microbial population that is distinct from traditional methods, enhancing the hydrolysis of pectin and other compounds. These compounds are then converted into flavor precursors and infused into the beans, yielding satisfactory results [17,25]. Moreover, this post-harvest technique offers beneficial uses in coffee production, including the standardizing of fermentation processes, prolongation of fermentation time, inhibition of fungal development, and enhancement of the overall quality of the coffee beverage. Particularly recognized for enhancing fruity attributes and generating distinct aromas [8,17,24,25], coffee fermentation also modulates the coffee’s aroma, resulting in a more varied spectrum of flavors [26,27].

Despite the advancements and innovations in coffee fermentation techniques, including self-induced anaerobic fermentation (SIAF), there remains a gap in understanding not only how spontaneous fermentation can be transformed into controlled fermentation, but also how fermentation processes are correlated with the development of unique flavor profiles in different coffee varieties. This gap highlights the need for more detailed research to optimize fermentation methods for enhancing specific sensory characteristics in coffee. In this context, this study aims to evaluate the effects of new fermentation processes on the organic, chemical, and sensory properties of fermented Arabica coffee fruits. Also, by using machine learning as a tool for data analysis and predictive modeling, specifically decision tree analysis, this study seeks to develop a decision-making framework that can potentially contribute to future research and assist coffee producers in enhancing their understanding of the performance of controlled fermentations, particularly through self-induced anaerobic fermentation (SIAF).

## 2. Materials and Methods

### 2.1. Coffee Fermentation Process and Experimental Design

*Coffea arabica* cv Catuaí Vermelho IAC-144, grown at an altitude of 1187 m on the Pântano farm, located in Coromandel, Minas Gerais State, Brazil (18°39′18″ S 46°50′53″ W), underwent fermentation using the SIAF method in a closed bioreactor. The processing of the coffee fruits was carried out on the same day as the harvest and included washing with water, subsequent removal of impurities, and classification according to the maturation stage. In the washer or hydraulic separator, based on density, coffee beans that float on water (dry, malformed, and immature) are separated from the heavier fruits (cherries and greenish beans).

For the fermentation, non-toxic polypropylene bioreactors with 200 L capacity were utilized. These bioreactors, previously sanitized with 5% peracetic acid, were sealed with a screw cap equipped with an airlock. This setup allowed the carbon dioxide generated during fermentation to escape while preventing outside air from entering. The bioreactors were employed for both natural and mechanically pulped coffee fermentation.

**Figure 1:**
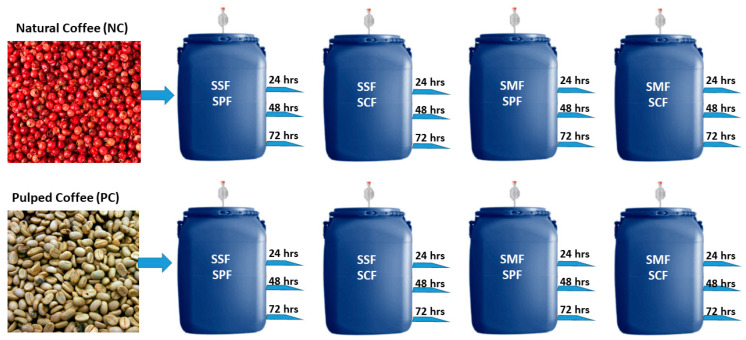
Experimental setup illustrating the fermentation process for natural coffee (NC) and pulped coffee (PC). Each type of coffee, natural coffee (NC) and pulped coffee (PC), was subjected to one of two fermentation methods: solid-state fermentation (SSF) or submerged fermentation (SMF), and within these methods, either spontaneous fermentation (SPF) or starter culture fermentation (SCF) was applied. These processes were carried out over three different time periods: 24, 48, and 72 h, creating a total of 24 distinct fermentation scenarios.

The detailed process for each treatment is illustrated in Figure 1 and further elaborated in Table S1 of the Supplementary Material. These treatments incorporated several processing variables, including the type of fruit treatment (natural, involving whole fruits, or pulped, involving mechanical removal of the skin); the addition of water (solid-state fermentation—SSF, without adding water, or submerged fermentation—SMF, with the addition of water, comprising 30% of the volume); and the type of microbiota (spontaneous fermentation using endogenous microbiota or the use of starter cultures, specifically *Saccharomyces cerevisiae*). For each process (natural coffee—NC, and pulped coffee—PC), a control sample was collected at the beginning (0 h) involving direct drying of the fruits without undergoing any controlled fermentation process. After sample collection and data recording, the coffee beans were thoroughly washed and spread out on raised beds for drying.

For the experiments that involved the use of a commercial inoculant, specifically Saccharomyces cerevisiae yeast, the manufacturer’s recommendations on the packaging were followed for its dilution. Concentration was adjusted to 10^9^ cells/mL, with an inoculum of 10% used in each bioreactor. Fermentations were then conducted over durations of 24, 48, and 72 h. At the conclusion of each fermentation period, samples were collected in triplicate for physicochemical analysis. To halt the fermentation process, the fermented coffee was rinsed with water and subsequently placed on raised beds for drying. This drying process continued until the beans reached a moisture content of approximately 12%. After drying, the beans were stored and allowed to rest for 25 days before proceeding with the coffee processing steps.

### 2.2. Chemical Analysis

#### 2.2.1. On-Farm Analysis

Five replicates of each analysis were performed both at the beginning (time 0 h) and at the end of each fermentation period (24, 48, and 72 h) during the coffee fermentation process. The soluble solids content, measured in degrees Brix, was determined using an optical digital refractometer, model MA888, from Akso^®^. Temperature and pH measurements were conducted using a pH meter for semi-solids, model pH in, also from Akso^®^. These analyses were carried out in accordance with the methods recommended by the AOAC [28].

#### 2.2.2. Analytical Determination of Biochemical Compounds in Fermented Coffee Beans

High-performance liquid chromatography (HPLC) was employed to determine the concentrations of sugars (sucrose, glucose, and fructose), alcohols (glycerol and ethanol), and organic acids (citric, malic, succinic, lactic, acetic, propionic, and butyric) in fruit samples at 0, 24, 48, and 72 h of fermentation. The analyses were conducted using an HPLC system, model LC-20A Prominence (Shimadzu Corp., Kyoto, Japan^®^), coupled with a photodiode array (PDA) detector and a refractive index (RID) detector at the Fermentation and Enzymatic Processes Laboratory of the Federal University of Uberlândia. Compound separation was achieved using a SUPELCOGEL C-610H column (30 mm × 7.8 mm) (Sigma Aldrich, St. Louis, MO, USA). The mobile phase consisted of 0.1% phosphoric acid, with an eluent flow rate of 0.5 mL/min for 35 min. The column oven was maintained at a temperature of 32 °C, and the injection volume was set at 20.0 μL. Organic acids were quantified using the PDA detector at 210 nm, while sugars and alcohols were determined using the RID. Data analysis and processing were conducted using LC-Solutions software (version 5.117 Shimadzu Corp., Kyoto, Japan), with quantification based on their respective calibration curves. To prepare the samples for analysis, 10 g of coffee beans was diluted in 100 mL of de-ionized water. To homogenize the mixture and facilitate the extraction of organic compounds, a domestic blender (Oster^®^ 1400W, São Paulo, SP, Brazil) was used for 2 min. The resulting slurry was filtered through two layers of polypropylene organza fabric. Following this initial filtration, the slurry was then centrifuged using a Heal Force (Neofuge^®^ 18R, Shanghai, China) at 13,000 rpm for 15 min at a temperature of 17 °C, and the supernatant was collected. Before injection, the supernatants underwent a second filtration using a 0.22 μm cellulose acetate filter (Sigma Aldrich, St. Louis, MO, USA) to purify the sample and preserve the integrity of the HPLC column. The prepared samples, in a volume of 20 μL, were injected directly into the chromatography column, following the procedure adapted by Elhalis et al. [10].

#### 2.2.3. Instrumental Texture

Texture profile analysis (TPA) of the coffee beans was conducted using a TA-XT2i Stable Micro System texturometer, focusing on determining hardness and fracturability parameters. A 25 mm diameter cylindrical aluminum probe, TPA macro—AIBCAKE 2, was utilized for this purpose. Data collection was performed using the ‘Texture Expert for Windows’ software, version 1.20 (Stable Micro Systems, Godalming, UK). For each sample, and corresponding to different storage times, individual green coffee beans were analyzed. Each bean was placed in the texturometer, and five measurements were taken per sample to assess the impact of storage time. During the tests, the probe was positioned 40 mm above the sample, with a return speed of 30 mm/second and a contact force of 30 g.

#### 2.2.4. Sensory Analysis

The coffee beans were sorted and roasted at the Dom Parillo roasting facility in Patos de Minas, Minas Gerais, Brazil, following the guidelines of the Specialty Coffee Association (SCA) [29]. Roasting was performed using an Atilla Gold Plus—10 Kg roaster (Atilla, Belo Horizonte, Brazil), with the beans being roasted no more than 24 h prior to tasting. Roasting duration varied between 8 and 12 min. Prior to sensory analysis, the beans were ground using an electric grinder (Tramontina, Carlos Barbosa, RS, Brazil). In order to assess the impact of fermentation duration on the final quality of the coffee beverage, roasted beans that had undergone 0, 24, 48, and 72 h of fermentation were prepared according to the SCA protocol [30]. For each sample, five cups were brewed, maintaining a precise ratio of 8.25 ± 0.25 g of coffee per 150 mL of water, with the water temperature controlled between 92.2 °C and 94.4 °C.

Five certified Q-Graders were tasked with evaluating the sensory attributes of the coffee samples. They assessed 10 specific attributes: fragrance/aroma, flavor, aftertaste, acidity, body, balance, uniformity, clean cup, sweetness, and overall quality, along with identifying defects. Each attribute was rated on a scale of 6 to 10, with increments of 0.25. Negative attributes, indicative of poor or undesirable flavors, were classified as defects and impacted the overall quality score of the coffee negatively. In addition to these quantitative assessments, the tasters also assigned descriptive sensory attributes to each sample, utilizing the framework provided by the flavor wheel [31].

### 2.3. Machine Learning Analyses and Multivariate Statistics

A machine learning-generated decision tree classification method was utilized to identify root attributes that most accurately classify subgroups in the study. MATLAB software, version 2023, was employed for this analysis, taking into account three key aspects: (i) the experimental design characteristics, (ii) results from high-performance liquid chromatography (HPLC), and (iii) sensory evaluation outcomes. To test and validate the accuracy of these decision trees, the ‘full-training-cross-validation’ (FTCV) method was applied. This method involves using all instances in the dataset simultaneously for both training and testing, with the outcome being the percentage of errors identified. To ascertain the influence of various chemical compounds on the fermentation process, a decision tree classification method was applied. This method utilized data from all measured variables in the study, forming a diagram that depicts the direct relationships between these sets of variables.

The data obtained from physicochemical and sensory analyses were statistically examined using the Tukey post hoc test for the comparison of means. Means with *p*-values less than 0.05 were considered statistically significant. Additionally, the datasets were analyzed using heatmap analysis for a visual representation of data patterns. Multivariate statistical analyses were performed by integrating datasets from both the physicochemical and HPLC platforms.

## 3. Results and Discussion

### 3.1. Physicochemical Composition

**Figure 2:**
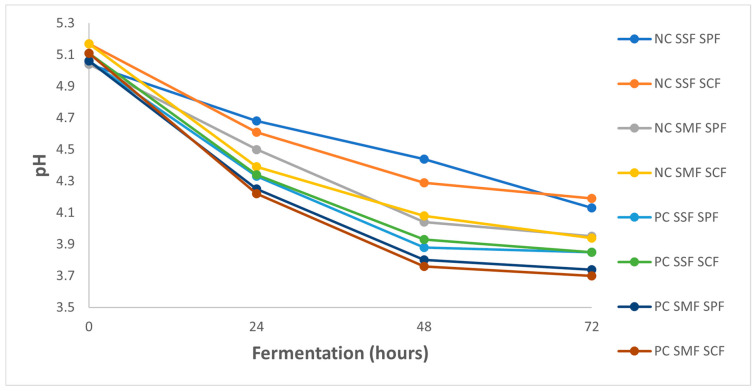
pH changes over a 72 h fermentation period for natural coffee (NC) and pulped coffee (PC) using different fermentation methods: solid-state fermentation (SSF) and submerged fermentation (SMF), each with either spontaneous fermentation (SPF) or starter culture fermentation (SCF).

The microbiota involved in coffee fermentation and the resulting biochemical reactions are complex [6]. This complexity is reflected in the variations in pH and temperature observed at the end of the fermentation process (Figure 2). In the case of Catuaí IAC 144 coffee, the fermentation commenced with an initial pH of 5.11 ± 0.10 in natural coffee and 5.08 ± 0.10 in pulped coffee, at a temperature of 21.5 ± 1.50 °C. After 24 h of fermentation, the PC treatment involving submerged fermentation with starter culture showed a notable decrease in pH value, recording at 4.22 ± 0.30. This decreasing trend in pH continued such that after 72 h, the lowest observed pH was 3.70 ± 0.10. During the fermentation process, the pH in the PC treatments generally decreased more compared to the NC treatments, though both followed similar behavior profiles. Notably, submerged fermentation treatments displayed a more pronounced reduction in pH value compared to solid-state fermentation treatments.

These findings align with the results of previous studies. Peñuela-Martínez et al. [9] reported that the initial pH of the Castillo variety of Arabica coffee fruits remained close to 5.25, with a final pH value between 3.7–4.0. Similarly, Braga et al. [32] observed comparable pH reduction behavior during the SIAF fermentation process of red Icatu coffee fruits over a period of 6 to 8 days.

pH value and titratable acidity are key indicators of coffee’s acidity, as the perceived acidity in coffee results from the donation of protons from acids to the taste receptors on the human tongue [33]. In this study, it was observed that the pH values of all fermented coffee beans were lower than those of unfermented beverages (0 h), suggesting that coffee made from fermented beans could exhibit relatively higher acidity.

**Figure 3:**
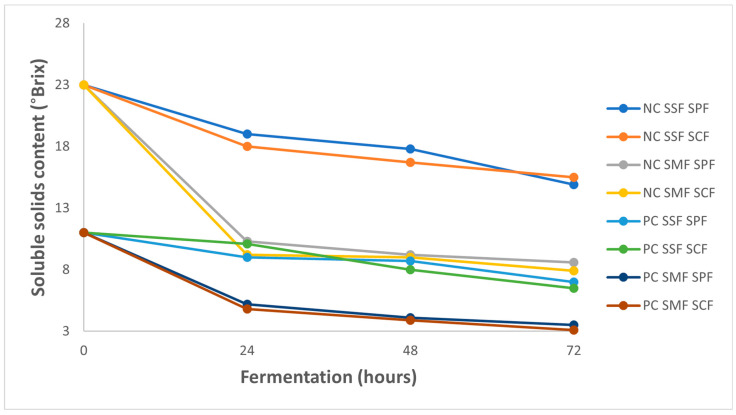
Soluble solids content (°Brix) changes over a 72 h fermentation period for natural coffee (NC) and pulped coffee (PC) using different fermentation methods: solid-state fermentation (SSF) and submerged fermentation (SMF), each with either spontaneous fermentation (SPF) or starter culture fermentation (SCF).

Consistent with expectations, there was a noticeable decrease in soluble solids (measured in degrees Brix) throughout the 72 h fermentation period (Figure 3). This pattern aligns with the reduction in sucrose levels observed within the fermenting mass over time. A decline in soluble solids content is a definitive sign of active fermentation processes, which metabolize sugars (sucrose, glucose, and fructose) and produce compounds such as organic acids (citric, malic, succinic, lactic, acetic, propionic, and butyric), alcohols (glycerol and ethanol), and esters, affecting both the flavor profile and chemical characteristics of the coffee [17,34,35].

Initially, the average Brix value for natural coffee was 23.65% ± 1.21, contrasting with 7.33% ± 1.90 for pulped coffee. These values were measured daily from the beginning (time zero) to the end of the fermentation period at 72 h. All treatments exhibited a decreasing trend in Brix levels, though the rate of decrease varied. This observation is consistent with findings by Bae, Haile, and Kang [18], who noted a similar reduction in Brix during the fermentation of Arabica coffee pulp over a 10-day period.

The type of fruit and the processing method significantly influenced the reduction in soluble solids content, in contrast to the type of microbiota present. On average, fermentation resulted in a 30–40% decrease in soluble solids content for SSF treatments, while SMF treatments experienced a reduction of 60–65%. This greater reduction in SMF treatments is likely due to the dilution effect caused by the presence of water, which decreases the concentration of soluble solids within coffee beans.

The fermentation process induces significant changes, particularly in pH and soluble solids content. These changes are driven by the activity of microorganisms, primarily bacteria and yeast, that metabolize sugars and other compounds in the coffee mucilage. This metabolic action leads to the production of various secondary metabolites such as organic acids (like lactic, butyric, and acetic acids) and the absorption of basic amino acids, resulting in a substantial reduction in pH [34,35,36]. Yeasts, as initial metabolizers of sugars in coffee pulp, play a crucial role in the complex biochemical transformations occurring within the coffee mucilage during fermentation [15]. Monitoring these changes is essential, as they significantly influence the kinetics of the fermentation process.

In addition to the changes in pH and soluble solids, it was observed that the fermentation process did not significantly alter the hardness parameters of the beans (Table S2). The textural characteristics of the coffee beans, such as force and hardness, remained relatively unchanged regardless of the fermentation treatment, indicating that these aspects of bean quality are not notably affected by the fermentation conditions.

### 3.2. Profile of Biochemical Compounds in Fermented Coffee Beans

#### 3.2.1. Sugars

**Figure 4:**
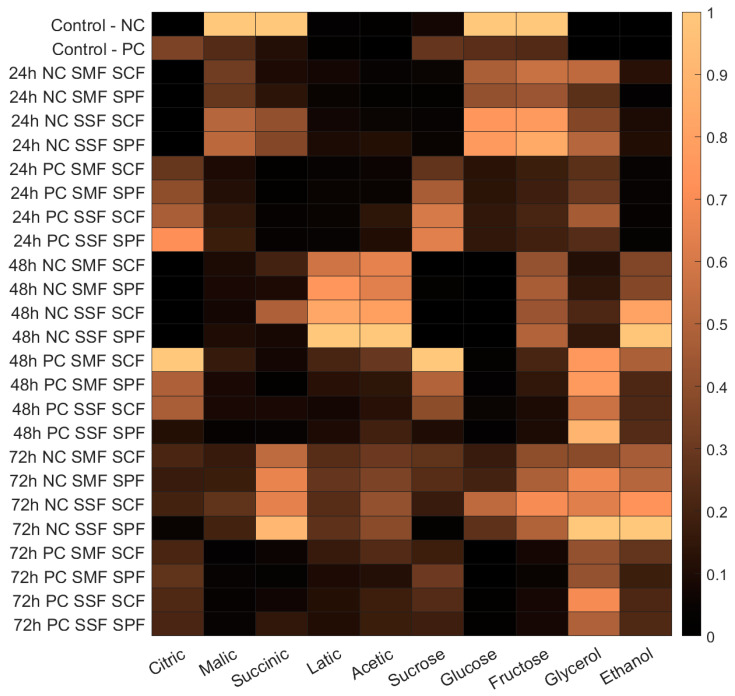
Normalized concentration heatmap of biochemical compounds in fermented coffee. The data illustrate the relative concentration levels of citric, malic, succinic, lactic, and acetic acids, as well as sugars (sucrose, glucose, and fructose), glycerol, and ethanol in natural coffee (NC) and pulped coffee (PC) samples. The concentrations are normalized against the maximum values observed for each compound. Fermentation processes include solid-state fermentation (SSF), submerged fermentation (SMF), spontaneous fermentation (SPF), and starter culture Fermentation (SCF), executed over 24, 48, and 72 h.

At the beginning of the fermentation process, a notable concentration of glucose, fructose, and sucrose was observed. Specifically, in NC, the concentrations were 41.43 ± 4.86 mg/g for glucose, 68.77 ± 6.19 mg/g for fructose, and 1.42 ± 0.45 mg/g for sucrose. In PC, the corresponding values were 11.41 ± 3.24 mg/g for glucose, 17.62 ± 3.37 mg/g for fructose, and 5.62 ± 1.45 mg/g for sucrose. Remarkably, the concentration of fructose at the beginning of fermentation was about twice that of glucose. Additionally, the levels of both glucose and fructose varied significantly depending on the type of coffee fruit (NC or PC), as well as the different fermentation methods: solid-state fermentation (SSF) and submerged fermentation (SMF), each with either spontaneous fermentation (SPF) or starter culture fermentation (SCF). The levels of these biochemical compounds in fermented coffee beans normalized against the maximum values observed for each compound are shown in Figure 4.

The peel and mucilage of coffee fruits are known to have a higher sugar content. Ribeiro et al. [37] highlighted that the concentrations of glucose and fructose are influenced by the processing method and tend to decrease from the beginning to the end of the fermentation process. This trend can be attributed to the composition of coffee beans, which are rich in polysaccharides like cellulose. These polysaccharides undergo hydrolysis during fermentation [38].

Residual sugars are a common finding in spontaneous coffee fermentations across various countries, including Brazil, Australia, Colombia, and Ecuador. These residual sugars are typically associated with short fermentation cycles, usually between 24 and 36 h [10,22,39,40]. However, our results indicate that simply extending the fermentation duration does not necessarily ensure the complete consumption of all sugars in the mucilage by the endogenous microbiota, particularly fructose. Nevertheless, a significant reduction in glucose levels over time was observed, particularly in pulped coffee treatments with fermentation times of 72 h. This trend aligns with the understanding that glucose is the preferred substrate in most fermentative pathways [41], leading to its more pronounced reduction in coffee fruits during the fermentation process.

#### 3.2.2. Organic Acids

Sweetness and acidity are pivotal attributes in determining coffee quality, alongside bitterness and aroma [42]. Green coffee beans contain a variety of organic acids, including citric, malic, acetic, butyric, propionic, succinic, and lactic acids [42]. The fermentation process significantly modifies the profile of these organic acids in coffee fruits. In unfermented coffee, malic and succinic acid predominate, particularly in NC fruits. Meanwhile, citric acid is more prominent in PC fruits. Post fermentation, a broader spectrum of organic acids, namely citric, malic, lactic, and acetic acids, were detected across various treatments. The levels of these biochemical compounds in fermented coffee beans normalized against the maximum values observed for each compound are also shown in Figure 4, regarding the type of coffee fruit (NC or PC), as well as the different fermentation methods.

The predominant organic acids in treatments involving NC fruits included lactic, acetic, succinic, and malic acids, whereas citric acid was more prominent in treatments involving PC fruits. Notably, natural coffee demonstrated a higher production of acids (citric, malic, succinic, and lactic acids) compared to pulped coffee in the SIAF treatments studied. This finding aligns with research by Jimenez et al. [43], who observed the presence of acetic, citric, lactic, malic, and succinic acids at the end of the fermentation process (480 h), with a notable increase in lactic acid production. Their study also revealed that natural coffee exhibited greater acid production (citric, malic, succinic, and lactic acids) than pulped coffee in SIAF treatments, highlighting the impact of the fermentation process and coffee processing method on the acid profile of the final product.

- Citric acid

Citric acid, commonly abundant in citrus fruits like oranges and lemons, exhibits higher concentrations in PC compared to NC during fermentation. The peak concentration of citric acid was observed in 48 h fermentation treatments, followed by a gradual decrease in 72 h fermentations. Citric acid catabolism in certain microorganisms involves its conversion through oxaloacetate, releasing acetate, and ultimately producing pyruvate, which can be reduced to lactic acid [44,45]. As a crucial intermediate of the tricarboxylic acid (TCA) cycle in aerobic respiration, citric acid is primarily metabolized through this cycle [44,46]. Moreover, citric and malic acids, naturally present in green coffee, can act as precursors for other acid degradation products such as citriconic, glutaric, fumaric, and maleic acids [47].

At the onset of fermentation (0 h), the average concentration of citric acid was 1.15 ± 0.67 mg/g for PC fruits, in contrast to a mere 0.007 ± 0.01 mg/g for NC fruits. By the end of the fermentation period (72 h), a reduction in citric acid levels was observed, with PC treatments showing 0.700 ± 0.08 mg/g and NC fruits averaging 0.519 ± 0.26 mg/g. The 48 h SMF and SCF PC treatment exhibited the highest peak concentration, at 2.43 ± 0.85 mg/g. Similar trends were noted by Bressani et al. [23] in the fermentation of Yellow Bourbon coffee using S. cerevisiae, where they recorded a citric acid content of 2.08 ± 0.01 mg/g in pulped coffee fruits. Conversely, other studies reported lower citric acid values post fermentation, such as Jimenez et al. [43] with 7.29 g/kg for NC fruits and 4.04 g/kg for PC fruits, Osório et al. [38] with 9.24 g/kg in fermented coffee mucilage, and Elhalis et al. [10] with 18 g/kg for PC fruits.

- Malic acid

The observed trend in malic acid, similar to that of succinic acid, exhibited higher concentrations in the early stages of fermentation, particularly in NC fruits. This pattern can be partially attributed to malolactic fermentation, a process where some microorganisms convert malic acid into lactic acid and carbon dioxide in an equal molar ratio [48]. This conversion could explain the observed decrease in malic acid concentration and the concurrent increase in lactic acid levels. De Bruyn et al. [22] also noted changes in the organic acid profile of coffee mucilage during fermentation, including decreases in gluconic, malic, and quinic acids.

Initially, the concentration of malic acid was 8.51 ± 0.12 mg/g for NC and 2.17 ± 0.48 mg/g for PC controls. These average values decreased to 1.69 ± 0.36 mg/g for NC and 0.32 ± 0.09 mg/g for PC fruits during fermentation. Among the fermented coffee treatments, 24 h SSF and SCF NC samples showed the highest malic acid content, at 4.35 ± 0.40 mg/g. Other studies reported lower malic acid values post fermentation: Jimenez et al. [43] found 2.02 g/kg for NC fruit and 2.75 g/kg for PC fruit, Osorio et al. [38] reported 4.64 g/kg for PC fruits, and Bressani et al. [23] noted 2.21 ± 0.01 mg/g for NC fruit with *S. cerevisiae*. Elhalis et al. [10] found that malic acid content in grains decreased from approximately 4.00 g/kg at the start of fermentation to 2.00 g/kg at the end. Evangelista et al. [15] reported an initial malic acid concentration of 0.73 g/kg, which also decreased during the fermentation process, potentially due to its conversion into lactic acid [49].

- Succinic acid

Succinic acid, naturally produced during coffee processing, significantly influences the final product’s characteristics. It can be synthesized by microbes such as *Bacillus* spp., heterofermentative Lactobacillus, and yeasts [50]. Before fermentation (0 h), succinic acid levels were 0.49 ± 0.02 mg/g for NC and 0.08 ± 0.05 mg/g for PC. Post fermentation (72 h), these levels averaged 0.10 ± 0.17 mg/g for NC and 0.03 ± 0.01 mg/g for PC. The highest succinic acid concentration, at 0.48 ± 0.12 mg/g, was yielded by 72 h SSF and SPF NC treatments.

Comparatively, some studies reported higher succinic acid levels post fermentation. Bressani et al. [23] observed 13.68 mg/g for NC fruit fermented with *T. delbrueckii*, and Da Mota et al. [24] recorded 4.25 mg/g for PC and 0.82 mg/g for NC in SIAF fermentation. However, Jimenez et al. [43] found lower concentrations: 2.38 g/kg for NC fruits and 1.60 g/kg for PC fruits. Elhalis et al. [10] noted that succinic acid levels (0.2%) remained relatively stable throughout fermentation, with insignificant differences between grains subjected to spontaneous fermentation and those suppressed by yeast growth. Da Mota et al. [49] suggested that microbial activity aids in the translocation of this acid to the bean, potentially altering the coffee beverage’s sensory profile.

- Lactic acid

Lactic acid, initially present in small quantities in coffee, emerges as the predominant acid during fermentation. This increase is attributed to the decarboxylation of malic acid by lactic acid bacteria (LAB), leading to a notable decrease in malic acid [51]. Lactic acid production is strongly correlated (≥0.45) with microbes like Lactobacillus, Lactococcus, Candida, and Saccharomyces [52]. Its production is crucial for fermentation, aiding in medium acidification and suppressing undesirable microorganisms [11].

At the start of fermentation, lactic acid concentrations were 4.15 ± 0.59 mg/g in NC and 2.77 ± 0.88 mg/g in PC. By 72 h, these levels rose dramatically to 52.20 ± 3.14 mg/g for NC and 25.05 ± 4.4 mg/g for PC. The highest concentration, 199.42 ± 38.85 mg/g, was observed in the 48 h SSF SPF NC treatment.

These findings align with those of Jimenez et al. [43], who reported 12.15 g/kg of lactic acid in NC and 4.89 g/kg in PC, and Evangelista et al. [15] who found 2.33 g/kg. Bae, Haile, and Kang [18] also identified lactic acid as the main acid present during fermentation. However, other studies reported lower end-of-fermentation values: Osório et al. [38] found 0.22–0.35 g/L in PC, Da Silva Vale et al. [52] recorded 5.06 ± 0.02 g/L in PC bioreactor layers, Bressani et al. [23] observed 0.11 ± 0.00 mg/g in PC, Da Mota et al. [24] reported 2.533 mg/g in NC and 2.136 mg/g in PC, and De Oliveira Junqueira et al. [39] noted 0.37 g/L in PC. The dominance of lactic acid post-fermentation is linked to the presence of LAB, with genera such as Weissella, Leuconostoc, and Lactobacillus identified by Evangelista et al. [15], and an increased abundance of Lactobacillus observed by Zhang et al. [53] post-fermentation.

- Acetic acid

Acetic acid, a key component in coffee fermentation, exhibits substantial increases after 24 h and peaks at 48 h, followed by a decline in 72 h treatments. Initial levels were 0.18 ± 0.10 mg/g in NC and 0.08 ± 0.06 mg/g in PC. The highest concentration, observed in 48 h SSF and SPF NC treatments, was 10.39 ± 0.88 mg/g. By the end of fermentation, the levels averaged 3.45 ± 0.56 mg/g for NC and 1.95 ± 0.78 mg/g for PC.

This reduction in acetic acid post 48 h can be attributed to the decline in Acetobacteraceae and Enterobacteraceae populations, overtaken by Lactobacillus, as reported by De Oliveira Junqueira et al. [39]. Elhalis et al. [10] noted that yeast-driven fermentations increase concentrations of various compounds, including acetic acid. Chindapan et al. [54] suggest that acetic acid contributes to coffee’s acidity and can impart fruity or unpleasant flavors depending on its concentration. Liu et al. [55] found that treating green coffee beans with acetic acid significantly enhances the final product’s quality.

Regarding sensory properties, acetic acid contributes to the sourness characteristic of coffee. It shares this trait with other acids like citric, formic, malic, quinic, pyruvic, succinic, fumaric, tartaric, and lactic acid, though each has unique aroma qualities. For instance, acetic acid has a vinegar-like aroma, pyruvic acid a burnt caramel flavor, and formic acid a pungent, fermented aroma [56].

In terms of comparative studies, Jimenez et al. [43] reported 6.04 mg/g of acetic acid in NC post fermentation, and Da Silva Vale et al. [52] recorded 1.00 ± 0.11 g/L in PC. Elhalis et al. [10] observed higher acetic acid levels in fermentations where yeast growth was suppressed, almost doubling in concentration.

- Butyric and propionic acids

Butyric and propionic acids were not detected in both the initial and final stages of fermentation across all treatments in this study. These acids are often associated with undesirable flavors in coffee [15]. Butyric acid, known for its rancid or buttery taste, can detrimentally affect the flavor profile of coffee. Its absence in coffee indicates that the fermentation process successfully prevented the growth of bacteria, such as specific Clostridium species, that are known to produce this acid. This careful management of the fermentation process is crucial in ensuring a desirable flavor profile in the final coffee product. Propionic acid, known for its pungent and rancid odor with a moderate cheese-like taste, can influence the flavor profile of fermented coffee. Its absence in coffee can significantly impact the overall sensory experience.

#### 3.2.3. Alcohols

Glycerol and ethanol were not initially present at the start of fermentation (0 h), but their levels increased progressively, peaking after 48 h. The 48 h SSF and SPF PC and 72 h SSF and SPF NC treatments had the highest glycerol concentrations, at 0.86 ± 0.01 mg/g and 0.84 ± 0.04 mg/g, respectively. Similarly, ethanol levels in these treatments reached 0.87 ± 0.01 mg/g and 0.84 ± 0.05 mg/g.

Comparatively, Da Mota et al. [24] reported ethanol levels of 5.451 mg/g for NC and 2.047 mg/g for PC, with glycerol at 0.365 mg/g for NC and 0.304 mg/g for PC after 22 h of fermentation. Elhalis et al. [10] detected glycerol at 24 h with a concentration of 0.9%, which then rose to 1.2% by the end of fermentation.

Glycerol production is enhanced under anaerobic conditions, acting as a protective agent for yeast against high osmotic pressure (leading to water efflux from the microbial cell), assisting in the maintenance of cellular redox balance (negative oxygen potential for anaerobic microorganisms), and supplying precursors for phospholipid synthesis (cell membrane components) [57]. Both glycerol and ethanol are valued in various beverages; glycerol contributes to the body and fullness in beer, wine, and spirits, while ethanol enhances palate fullness in wine [58].

#### 3.2.4. Analysis of Organic Compounds from Fermentation by Machine Learning

The decision tree classification method was employed to discern the influence of various factors on the production of chemical compounds during the fermentation of coffee. This methodology considered variables such as (i) fermentation time, (ii) type of fruit, (iii) addition of water, and (iv) type of microbiota to establish the direct relationships between these variables and their influence on the production of chemical compounds. Each chemical compound was categorized into two groups (high and low) based on the median of the group, ensuring an equal number of occurrences in each group. Thus, it can be understood how predictions can be made regarding the quantity of chemical compounds in relation to different fermentative processes. For citric acid, values equal to or above 0.59 mg/g were categorized as high, while those below this threshold were considered low. This categorization was similarly applied to succinic acid (0.05 mg/g), glycerol (0.40 mg/g), ethanol (5.03 mg/g), acetic acid (1.52 mg/g), lactic acid (20.68 mg/g), and malic acid (1.157 mg/g).

**Figure 5:**
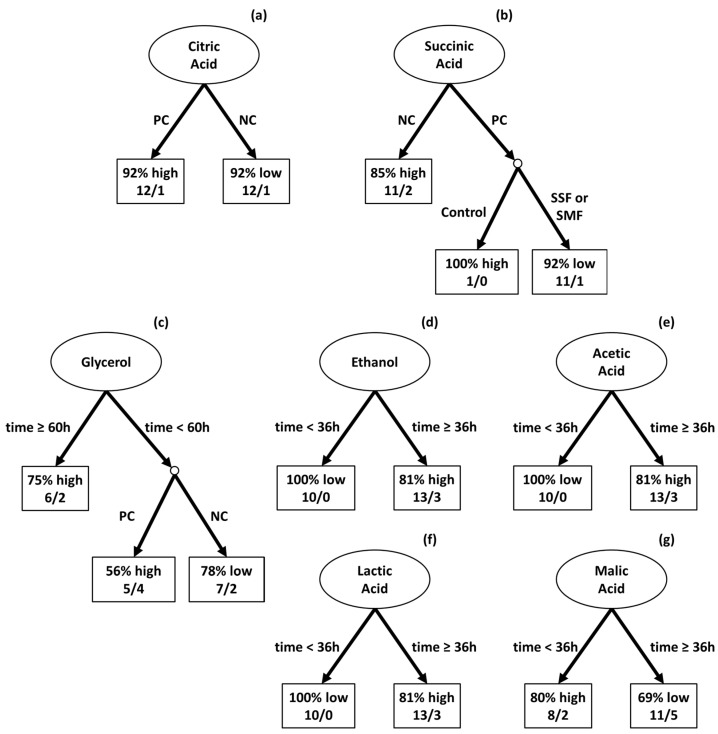
machine learning-generated decision tree for biochemical compound production during coffee fermentation. These decision trees classifies specific compounds—citric acid (**a**), succinic acid (**b**), glycerol (**c**), ethanol (**d**), acetic acid (**e**), lactic acid (**f**), and malic acid (**g**)—into ‘high’ or ‘low’ groups based on the median concentration levels identified within the data. Percentages reflect the likelihood of a compound falling into the high or low category, while ratios indicate the number of instances that correspond to each classification.

Time was identified as a primary factor influencing the production of most organic compounds. However, there were notable exceptions. In the case of citric acid production (Figure 5a), the type of coffee fruit (PC or NC) was more influential. PC fruits favored higher citric acid concentrations during fermentation, whereas NC fruits led to greater succinic acid production (Figure 5b). Additionally, specific conditions were found to be crucial for the optimal concentrations of certain compounds: glycerol production (Figure 5c) was most significant with fermentation durations of 60 h or more; ethanol (Figure 5d), acetic acid (Figure 5e), and lactic acid (Figure 5f) required at least 36 h; and for malic acid (Figure 5g), a fermentation period shorter than 36 h was preferable.

### 3.3. Sensory Analysis

**Figure 6:**
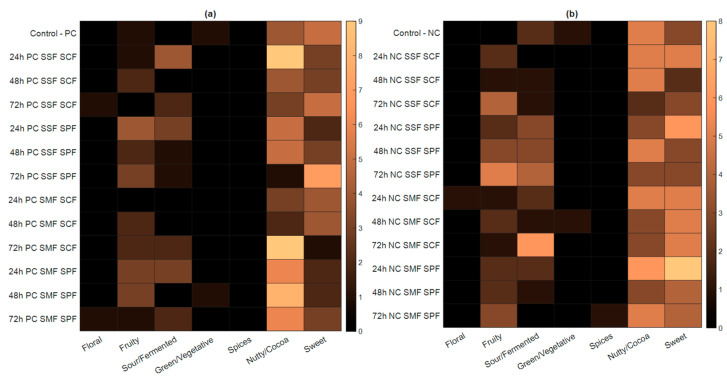
Number of descriptive coffee attributes provided by the Q-graders for the different fermentation processes for NC (**a**) and PC (**b**), where SSF = solid-state fermentation, SMF = submerged fermentation, SPF = spontaneous fermentation, and SCF = starter culture fermentation.

The market for high-quality coffee, recognized as a specialty product, continues to expand annually [59]. Sensory descriptors, defined according to the coffee taster flavor wheel [31], highlight the distinct sensory characteristics that emerge from different post-fermentation methods. In this study, five Q-Graders evaluated the coffee samples using the Specialty Coffee Association (SCA) cupping protocol [30]. All coffee samples scored above 80, categorizing them as ‘Specialty Coffees’. The various descriptive coffee attributes provided by the Q-graders for the different fermentation processes are shown in Figure 6 for PC (a) and NC (b). It is noticed that fermentation yielded beverages with greater complexity compared to the control treatment.

In terms of flavor descriptors, all samples featured notes of chestnut and caramel/molasses. However, there was a variation in descriptor complexity across the different samples (Table S3). The primary attributes consistently identified in all samples, irrespective of the processing method, were nutty/cocoa, fruity, sweet, and fermented. It is known that anaerobic fermentation can impart a fruitier profile to coffee, but the specific fruit descriptors vary depending on the fermentation process technique [17,25].

The observed variations in coffee flavor can predominantly be attributed to the different kinds of fermentations analyzed. This phase involves microbial activity from different treatments, leading to the production of a diverse array of metabolites. These metabolites diffuse into the green coffee beans and are retained even after roasting, significantly influencing the chemical composition of the processed coffee. This change in chemistry plays a crucial role in determining the quality of the final coffee preparation [60]. Different fermentation processes result in distinct chemical compositions, giving the evaluated coffee beverages unique sensory properties. These variations cater to and satisfy diverse market preferences [6].

Despite the results obtained, it was not possible to establish a direct correlation between the chemical changes and their sensory implications, as such analysis involves other factors not examined in this study, such as the volatile compounds and microbiota involved in the fermentation process, among others.

#### Sensory Analysis of Fermented Coffees Using Artificial Intelligence

**Figure 7:**
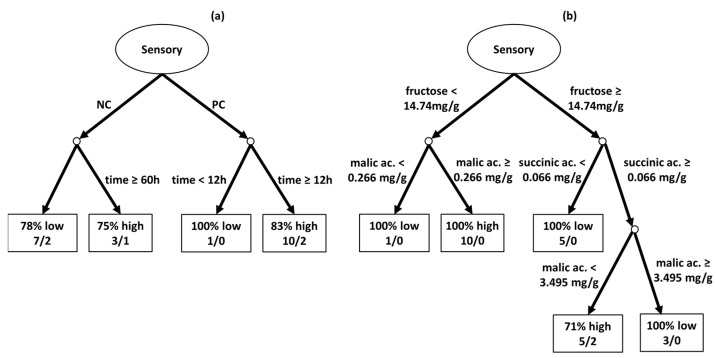
Machine learning-generated decision tree for sensory scores using the different fermentation scenarios as predictors in (**a**) and using biochemical compounds as predictors in (**b**). Q-Grader scores were divided into ‘high’ for scores above 83 points and ‘low’ for scores of 83 or below.

To analyze the relationship between various processing methods and sensory evaluation scores, a decision tree was developed using machine learning techniques. The scores given by Q-Graders were categorized into two groups (high and low) so that each group had a roughly equal number of records. Scores exceeding 83 points were designated as high (15 records), while scores at or below 83 points were considered low (11 records). The resulting decision tree, depicted in Figure 7a, demonstrates an overall accuracy exceeding 80%. The tree reveals that the type of fruit was the most influential factor in classifying the processes, as it forms the initial branch of the decision tree.

Analyzing the second level of decision-making in the tree for natural coffees (Figure 7a), it becomes apparent that fermentation time played a crucial role in differentiating between high and low scores. Processes with fermentation times beyond 60 h achieved high scores in 75% of instances, whereas those with shorter fermentation times had low scores 78% of the time. For pulped coffees, the other branch of the tree, fermentation time was again a significant determinant. Here, any fermentation lasting more than 12 h significantly increased the likelihood (83%) of attaining a higher score compared to coffees without fermentation.

Another investigation focused on the interplay between the average concentrations of organic compounds and sensory evaluation scores, as illustrated in Figure 7b. The 83-point mark was again employed as the dividing line between high and low scores, and this decision tree achieved an overall accuracy of 92%. In this analysis, fructose emerged as the most critical variable. Low fructose levels (below 14.74 mg/g) combined with high malic acid levels (0.266 mg/g or more) were strongly associated with high scores, as evidenced in 10 distinct experimental settings. Conversely, high fructose concentrations (at least 14.74 mg/g) paired with low succinic acid levels (below 0.066 mg/g) typically resulted in low scores in five experiments. Additionally, high fructose levels coupled with high succinic acid concentrations (0.066 mg/g or more) necessitated malic acid levels below 3.495 mg/g to achieve high scores in 71% of cases. Thus, the precise balance of organic compounds generated during the fermentation process is crucial for achieving favorable sensory evaluations of coffee.

## 4. Conclusions

The fermentation process has been shown to significantly enhance the sensory quality and descriptive profile of coffee, adding value to the product. Utilizing closed bioreactors in the SIAF method notably promotes the production of lactic and acetic acids, as well as ethanol, in coffee. Both the type of coffee, NC or PC, and the processing method, SSF or SMF, were determinants in the levels of organic compounds produced. Specifically, NC demonstrated a greater production of certain acids (malic, succinic, and lactic acids) than PC when processed using the SIAF method. The SMF process was more conducive to higher concentrations of lactic acid, whereas the SSF process favored increased ethanol content. Additionally, malic and succinic acids and fructose were found to be key factors influencing the sensory scores of fermented coffee samples. It is noteworthy that the fermentation process did not alter the textural properties of the coffee beans. Thus, the fermentation of coffee fruits significantly alters their chemical composition and results in the development of unique sensory attributes.

When applying machine learning techniques (decision tree analysis) to the results of different fermentative coffee processing methods, it was possible to provide a decision-making framework with the potential to contribute to future research and assist coffee producers in accumulating knowledge about the performance of controlled fermentations via SIAF. This can lead to improvements in coffee quality and the production of specialty coffees with diverse chemical and sensory profiles. Therefore, it is recommended that producers conduct extensive testing to identify the optimal processing time, as the outcome is influenced by multiple factors. These include coffee variety, its chemical composition, the inherent microbial community of the coffee, fermentation conditions, and other variables.

## Data Availability

Data is contained within the article and supplementary materials.

## Supplementary Materials

The following supporting information can be downloaded at: https://www.mdpi.com/article/10.3390/foods13030454/s1, Table S1: Treatments conducted in the coffee fermentation process; Table S2: Texture analysis averages (strength) for the treatments analyzed; Table S3: General sensory score and descriptive attributes of coffee beans during the coffee fermentation process.
