# Supplementary material for: Evaluation of Arabica Coffee Fermentation Using Machine Learning
Source: Foods. 2024 Feb 1;13(3):454. doi: 10.3390/foods13030454 (PMC10855612; doi:10.3390/foods13030454)
Supplement: Supplementary file 1 [file foods-13-00454-s001.zip › Suplementar Material S1.pdf]

Supplementary Material

Table S1. Treatments conducted in the coffee fermentation process.

Type of fruit[1]	Water adding[2]	Type of microbiota[3]	Fermentation hours
NC	Control	Control	0
PC	Control	Control	0
NC	SSF	SPF	24
			48
			72
NC	SSF	SCF	24
			48
			72
NC	SMF	SPF	24
			48
			72
NC	SMF	SCF	24
			48
			72
PC	SSF	SPF	24
			48
			72
PC	SSF	SCF	24
			48
			72
PC	SMF	SPF	24
			48
			72
PC	SMF	SCF	24
			48
			72

[1]NC= Natural coffee; PC= Pulped coffee

[2]SSF= Solid-state Fermentation; SMF = Submerged Fermentation

[3]SPF = Spontaneous fermentation; SCF= Starter culture fermentation
