# Supplementary material for: Evaluation of Arabica Coffee Fermentation Using Machine Learning
Source: Foods. 2024 Feb 1;13(3):454. doi: 10.3390/foods13030454 (PMC10855612; doi:10.3390/foods13030454)
Supplement: Supplementary file 1 [file foods-13-00454-s001.zip › Suplementar Material S2.pdf]

Table S2. Texture analysis averages (strength) for the treatments analyzed.

Treatment	Mean	Standard deviation
Control - NC	53392[a]	4738
Control - PC	50060 [a]	5115
24h NC SSF SPF	56683 [a]	2917
24h NC SSF SCF	48102 [a]	5571
24h NC SMF SPF	48618 [a]	2674
24h NC SMF SCF	52887 [a]	3665
24h PC SSF SPF	49598 [a]	4660
24h PC SSF SCF	49019 [a]	5196
24h PC SMF SPF	47907 [a]	4695
24h PC SMF SCF	52384 [a]	3859
48h NC SSF SPF	50521 [a]	4735
48h NC SSF SCF	49004 [a]	3122
48h NC SMF SPF	50110 [a]	4454
48h NC SMF SCF	50836 [a]	3025
48h PC SSF SPF	53819 [a]	3859
48h PC SSF SCF	51554 [a]	5763
48h PC SMF SPF	49524 [a]	1704
48h PC SMF SCF	50454 [a]	4348
72h NC SSF SPF	52660 [a]	2622
72h NC SSF SCF	46902 [a]	2531
72h NC SMF SPF	51054 [a]	5030
72h NC SMF SCF	51870 [a]	3738
72h PC SSF SPF	51380 [a]	4272
72h PC SSF SCF	47522 [a]	4331
72h PC SMF SPF	51806 [a]	4036
72h PC SMF SCF	47557 [a]	2919

NC= Natural coffee; PC= Pulped coffee

SSF= Solid-state Fermentation; SMF = Submerged Fermentation

SPF = Spontaneous fermentation; SCF= Starter culture fermentation

Data are shown as the mean. ± = standard deviation. Different lowercase letters indicate a statistically significant difference ($p < 0.05$) by the Tukey test evaluating the interaction of strength data with fermented coffee treatments.
