# Supplementary material for: Evaluation of Arabica Coffee Fermentation Using Machine Learning
Source: Foods. 2024 Feb 1;13(3):454. doi: 10.3390/foods13030454 (PMC10855612; doi:10.3390/foods13030454)
Supplement: Supplementary file 1 [file foods-13-00454-s001.zip › Suplementar Material S3.pdf]

Table S3. General sensory score and descriptive attributes of coffees during the coffee fermentation process.

Treatment	Overall score	Descriptive sensory attributes
Control - NC	82.40 ±0.75	Caramelized, nutty, chocolate.
Control - PC	82.50 ±0.62	Caramelized, nutty, chocolate.
24h NC SSF SPF	82.40 ±0.57	Caramelized, nutty, chocolate, citrus fruit, brown sugar, citric acid.
24h NC SSF SCF	82.95 ±0.12	Caramelized, nutty, leve frutado, brown sugar.
24h NC SMF SPF	83.45 ±0.57	Caramelized, nutty, chocolate, molasses.
24h NC SMF SCF	82.25 ±0.23	Caramelized, nutty, chocolate, citric acid.
24h PC SSF SPF	84.30 ±1.20	Caramelized, nutty, chocolate, citric acid.
24h PC SSF SCF	83.15 ±0.42	Caramelized, nutty, chocolate, citric acid.
24h PC SMF SPF	83.30 ±0.79	Caramelized, nutty, chocolate, berry, brown sugar, citric acid.
24h PC SMF SCF	83.15 ±0.68	Caramelized, nutty, chocolate, molasses, sour.
48h NC SSF SPF	83.00 ±0.62	Caramelized, nutty, chocolate, brown sugar, citric acid.
48h NC SSF SCF	82.70 ±0.77	Caramelized, nutty, chocolate, citrus fruit, citric acid.
48h NC SMF SPF	82.95 ±0.12	Caramelized, nutty, citrus fruit, brown sugar.
48h NC SMF SCF	83.35 ±0.31	Caramelized, nutty, berry, citric acid.
48h PC SSF SPF	83.30 ±0.54	Caramelized, nutty, chocolate, citric acid.
48h PC SSF SCF	83.75 ±1.23	Caramelized, nutty, chocolate, berry.
48h PC SMF SPF	83.15 ±0.72	Caramelized, nutty, chocolate, berry.
48h PC SMF SCF	82.60 ±0.83	Caramelized, nutty, chocolate.
72h NC SSF SPF	83.50 ±0.54	Caramelized, nutty, chocolate, citric acid.
72h NC SSF SCF	84.50 ±0.92	Caramelized, nutty, brown sugar.
72h NC SMF SPF	82.60 ±0.64	Caramelized, nutty, citrus fruit.
72h NC SMF SCF	83.35 ±1.12	Caramelized, nutty, brown sugar, citric acid.
72h PC SSF SPF	83.60 ±0.35	Caramelized, nutty, molasses, citrus fruit, citric acid.
72h PC SSF SCF	84.25 ±1.28	Caramelized, nutty, brown sugar, citrus fruit, malic acid..
72h PC SMF SPF	83.15 ±1.25	Caramelized, nutty, chocolate, berry, citric acid.
72h PC SMF SCF	83.00 ±0.75	Caramelized, nutty, chocolate, berry, citric acid.

NC= Natural coffee; PC= Pulped coffee

SSF= Solid-state Fermentation; SMF = Submerged Fermentation

SPF = Spontaneous fermentation; SCF= Starter culture fermentation
